# Validation of a new fully automated software for 2D digital mammographic breast density evaluation in predicting breast cancer risk

**DOI:** 10.1038/s41598-021-99433-3

**Published:** 2021-10-06

**Authors:** Paolo Giorgi Rossi, Olivera Djuric, Valerie Hélin, Susan Astley, Paola Mantellini, Andrea Nitrosi, Elaine F. Harkness, Emilien Gauthier, Donella Puliti, Corinne Balleyguier, Camille Baron, Fiona J. Gilbert, André Grivegnée, Pierpaolo Pattacini, Stefan Michiels, Suzette Delaloge

**Affiliations:** 1 Epidemiology Unit, Azienda Unità Sanitaria Locale – IRCCS di Reggio Emilia, Via Amendola 2, 42122 Reggio Emilia, Italy; 2 Section of Public Health, Department of Biomedical, Metabolic and Neural Sciences, Center for Environmental, Nutritional and Genetic Epidemiology (CREAGEN), University of Modena and Reggio Emilia, Via Università 4, 41121 Modena, Italy; 3 Predlife, Espace Maurice Tubiana, 39 rue Camille Desmoulins, 94800 Villejuif, France; 4 Division of Informatics, Imaging and Data Sciences, Faculty of Biology, Medicine and Health, Manchester Academic Health Science Centre, University of Manchester, Stopford Building, Oxford Road, Manchester, M13 9PT UK; 5 Prevent Breast Cancer and Nightingale Breast Screening Centre, Manchester Academic Health Science Centre, Manchester University NHS Foundation Trust, Southmoor Road, Wythenshawe, Manchester, M23 9LT UK; 6 Screening Unit, ISPRO - Oncological Network, Prevention and Research Institute, via Cosimo il Vecchio 2, 50139 Florence, Italy; 7 Medical Physics Unit, Department of Oncology and Advanced Technologies, Azienda Unità Sanitaria Locale – IRCCS di Reggio Emilia, Viale Umberto I 50, 42123 Reggio Emilia, Italy; 8 Clinical Epidemiology Unit, ISPRO - Oncological Network, Prevention and Research Institute, via Cosimo il Vecchio 2, 50139 Florence, Italy; 9 Department of Radiology, Institut Gustave-Roussy, 114 rue Edouard Vaillant, 94800 Villejuif, France; 10 UNICANCER, Institut Bergonié, 229, cours de l’Argonne CS 61283, 33076 Bordeaux Cedex, France; 11 Department of Radiology, NIHR Cambridge Biomedical Research Centre, University of Cambridge, Cambridge, CB2 0QQ UK; 12 Department of Radiology, Addenbrooke’s Hospital, Cambridge University Hospitals National Health Service Foundation Trust, Cambridge, CB2 0QQ UK; 13 Senology Unit, Institute Jules Bordet, Boulevard de Waterloo 121, 1000 Brussels, Belgium; 14 Department of Diagnostic Imaging, Azienda Unità Sanitaria Locale – IRCCS di Reggio Emilia, Viale Umberto I 50, 42123 Reggio Emilia, Italy; 15 Biostatistics and Epidemiology Service, Centre de Recherche en Epidémiologie et Santé des Populations, Gustave Roussy, Université Paris-Sud, 114, rue Edouard-Vaillant, 94805 Villejuif, France

**Keywords:** Breast cancer, Health care, Medical research, Oncology

## Abstract

We compared accuracy for breast cancer (BC) risk stratification of a new fully automated system (DenSeeMammo—DSM) for breast density (BD) assessment to a non-inferiority threshold based on radiologists’ visual assessment. Pooled analysis was performed on 14,267 2D mammograms collected from women aged 48–55 years who underwent BC screening within three studies: RETomo, Florence study and PROCAS. BD was expressed through clinical Breast Imaging Reporting and Data System (BI-RADS) density classification. Women in BI-RADS D category had a 2.6 (95% CI 1.5–4.4) and a 3.6 (95% CI 1.4–9.3) times higher risk of incident and interval cancer, respectively, than women in the two lowest BD categories. The ability of DSM to predict risk of incident cancer was non-inferior to radiologists’ visual assessment as both point estimate and lower bound of 95% CI (AUC 0.589; 95% CI 0.580–0.597) were above the predefined visual assessment threshold (AUC 0.571). AUC for interval (AUC 0.631; 95% CI 0.623–0.639) cancers was even higher. BD assessed with new fully automated method is positively associated with BC risk and is not inferior to radiologists’ visual assessment. It is an even stronger marker of interval cancer, confirming an appreciable masking effect of BD that reduces mammography sensitivity.

## Introduction

Mammographic breast density (BD) is the absolute amount or percentage of fibro glandular tissue in the breast. It is an established risk factor for breast cancer (BC) and an important determinant of screening sensitivity as it may hamper cancer detection in mammograms, i.e., the masking effect. Radiologist visual assessment, an area-based method, commonly used for judgment of breast density, is subject to several important limitations, such as high subjectivity and intra- and inter-observer variability^1^ resulting in low reliability and reproducibility^2,3^. In addition, breast density assessment performed by radiologists (using Breast Imaging-Reporting and Data System-BI-RADs)^4^ is time consuming and prone to poor reproducibility, making its application in organized screening challenging.

Consequently, there are important clinical and research repercussions. Breast density is being used to stratify women based on their breast cancer risk to decide if they need further imaging assessment in clinical trials, such as ultrasound or MRI^5,6^, or if they would benefit from a shorter mammography screening interval. Low reproducibility of mammographic density measures may result in conflicting recommendations and inaccurate risk stratification which could increase the probability of an interval BC.

The potential of personalized BC screening based on individual risk over universal “one-size fits all” screening has been recognized and several randomized trials have been launched to address this issue, such as Tailored Breast Screening Trial (TBST)^7^, WISDOM^8^ and My Personalized Breast Screening (MyPeBS)^9^. MyPeBS is an international randomized, multicentre study aimed to assess the effectiveness of a risk-based breast cancer screening strategy (using clinical risk scores and polymorphisms) compared to standard screening (according to current national breast cancer screening policies). BD evaluation plays a central role in the MyPeBS trial as mammographic density contributes to the risk assessment in the intervention arm and it determines whether women should receive supplemental ultrasound.

Several semi-automated and fully automated methods for reproducible assessment of breast density have been developed and evaluated^10–13^; these include approaches based on area-based and volumetric measures, and in some cases include mammographic pattern, however variations in assessment methodology make direct comparisons of risk prediction challenging^14–19^. DenSeeMammo (DSM) has been validated for density assessment and shows a higher degree of agreement than that reported in studies on other vendors’ automated volumetric assessment tools^20^. However, the software has not yet been validated for prediction of breast cancer risk and for assessing the risk of masking (i.e., the risk of having interval or subsequent round cancer instead of screen-detected cancer).

The primary aim of this study was to evaluate a recently developed automated system for breast density assessment (DenSeeMammo) and its ability to stratify breast cancer risk, by comparing discriminative accuracy for BC risk prediction to a non-inferiority threshold defined on the basis of visual assessment by radiologists. The secondary aim was to estimate how the fully automated BD assessment is able to measure the masking effect.

## Methods

### Study population

**Figure 1:**
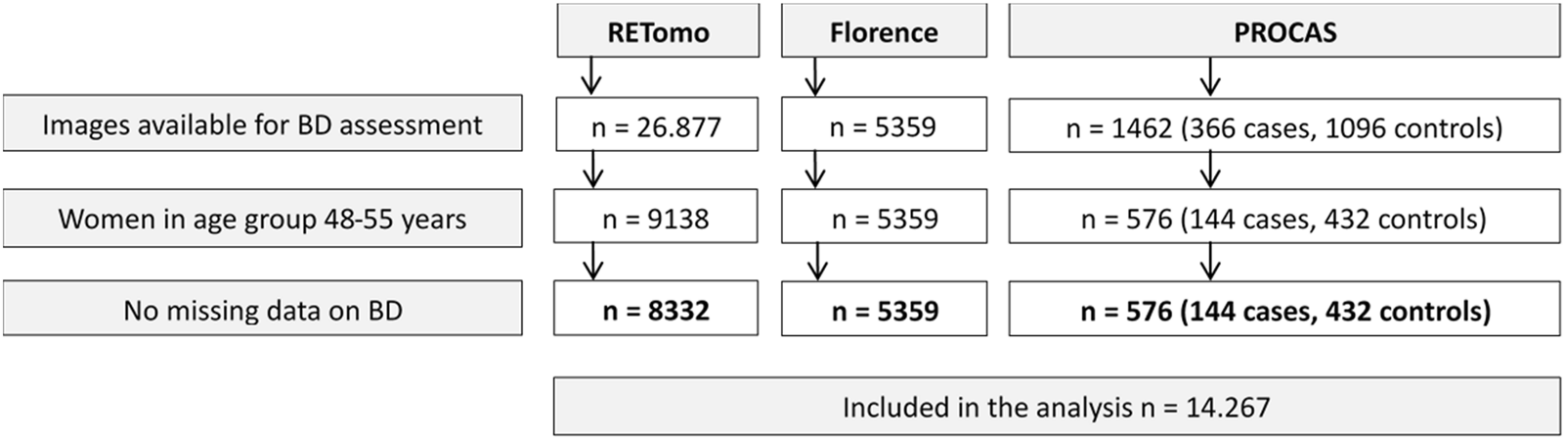
Flowchart of study participants. *BD* breast density, *FFDM* fool-field digital mammography.

This study represents a pooled analysis of images collected within the RETomo trial^21^, Florence study^17^ and PROCAS case–control study^18^, with a total of 14,267 women 48–55 years old, of whom 322 developed breast cancer. A summary of studies included is provided in Supplementary Table 1 while the flowchart of study participants is presented in Fig. 1.

#### Florence study

A cohort study including 15,952 women who had their first screening digital mammography aged 49–54 years old during the period 2006–2013. Women were routinely reinvited after 2 years, and the study follow-up lasted 2.5 years. Only 5359 2D full-field digital mammography images were available for breast density analysis since some images were performed with mammography equipment (GE 2000D) which is not supported by DenSeeMammo software.

#### RETomo (Reggio Emilia tomosynthesis) trial

A cohort study including more than 27,000 women aged 45–70 years old attending screening between March 2014 and July 2017 and who had already participated in at least one round of the Reggio Emilia screening program. Women aged 45–49 years were routinely reinvited after 1 year and women aged 50–74 years after 2 years; the study follow-up lasted 2.5 years. Of the 48–55 year old women density assessment was available in 8332 women.

#### PROCAS (predicting risk of cancer at screening) study

A cohort study initially including 44,658 women with full field digital mammograms and attending both first and subsequent screening rounds from October 2009 to March 2015. Women are routinely reinvited every 3 years. A subset of 576 women in whom breast cancer was detected at time of entry to the study plus controls (432 controls, 144 cancer cases) in the age range 48–55 years were available for breast density assessment with automated software in this study.

No exclusion criteria other than those that were study-specific were applied. For women who had several mammograms, the first mammogram was analysed. Pooled analysis was conducted on individual anonymized data.

For all three studies local Ethics Committee approval had been obtained and informed consent given by all woman participating in the relevant study. Ethics approval for the PROCAS was through the North Manchester Research Ethics Committee (09/H1008/81). ReTomo was approved by the provincial Ethical Committee (November 11, 2013; ASMN 2013/0029304). The Ethics Committee of the Florence district gave their approval for the Florence study on 12 September 2017 (n.11630_oss). All methods involving human participants were performed in accordance with the Declaration of Helsinki.

### Acquisition of images

Cranio-caudal (CC) and mediolateral oblique (MLO) pairs (left and right breast) of 2D full-field digital mammography (FFDM) of all women recruited were obtained from Picture Archiving and Communication Systems (PACS) and processed by DSM. Ten women were excluded as it was not possible to obtain suitable images. Images were from different vendors and systems (GE Essential and Siemens Mammomat Inspiration). Detailed description of image acquisition is provided in Supplementary Table S1.

### Breast density assessment

DenSeeMammo 1.2 (Predilife, Villejuif, France)—is a Food and Drug Administration (FDA) approved, fully automated software for assessing breast density providing a BI-RADS category density grade. DenSeeMammo handles processed ‘for-presentation’ images extracted from DICOM files as input (CC + MLO, or CC or MLO). It provides results on a per patient basis, using the maximum density category of the two breasts. The method is based on a comparison with databases containing images previously scored by the Mammography Quality Standards Act (MQSA) radiologists using BI-RADS 5th Edition^4^. With DenSeeMammo, all assessments are based on BI-RADS 5th Edition which takes into account percent density and density distribution in the breast to reflect risk of masking^4^. DenSeeMammo received 510(k) FDA clearance in 2017 and in 2018 for automatic breast density evaluation on GE and Hologic equipment.

### Endpoints

Cancers included in the analysis were: (1) prevalent cancers, i.e., cancers detected at inclusion to the study, (2) incident cancers i.e., cancer not detected at the study inclusion mammogram but occurred at a subsequent screening round including (3) interval cancers i.e., cancer diagnosed after a negative screening mammogram and before the next screening mammogram. The main endpoint is incident cancer (interval and screen-detected at the second round). We also report prevalent cases and all cases (incident and prevalent), since including only incident cancers in women undergoing screening overestimates the ability of density to predict cancer risk. A substantial proportion of undetected prevalent cancers in women with dense breasts at baseline become symptomatic or detectable later during follow-up, while in women with fatty breasts, they are more efficiently detected by the baseline mammography.

The main analysis was the Area Under the Receiver Operating Characteristic curve (AUC) for DSM software’s prediction of incident breast cancer risk, calculated from all the women for which we had available 2D mammography suitable for DSM assessment. This analysis was performed only on incident and interval cancers, because in its practical application in screening, risk stratification is meaningful only for women who have not been diagnosed with a cancer.

We also report the odds ratios for incident, interval, prevalent and all cancers. The association with all cancers may better reflect the strength of the aetiological link between density and breast cancer risk as it is not affected by the bias of excluding more efficiently prevalent cancers in fatty breasts than in dense breasts. The difference in the strength of association between prevalent and interval cancers can be interpreted as an indirect measure of the software’s ability to identify the masking effect of breast density: if the relative risk of having interval cancers in dense breasts vs. non-dense breasts is higher than the relative risk of having prevalent screen detected cancers, this means that the breast density assessment was able to identify part of the masking effect due to breast density.

### Statistical analysis

Descriptive statistics were used to present the distribution of women and cancer cases within centres and breast density categories. The association between breast density assessed by DSM and BC risk was estimated using logistic regression and odds ratios (OR) with 95% confidence intervals (95% CI). Due to the small number of cancers in BI-RADS A category, the sum of the two lowest BI-RADS categories (A and B) was considered as a reference category. To take into account the different study designs of the PROCAS case–control study and the other two cohort studies, for the analysis of risk of all cancers on pooled data, we applied a sampling probability weight to the controls of the PROCAS study in a ratio 1:15 compared to cancers, as estimated from the risk observed in the underlying cohort of the PROCAS study. Given that models adjusted for age and study centre yielded similar results as crude models, the results of the crude model are presented.

As a single measure of accuracy, i.e., ability of the automated system to discriminate between cases and controls, area under the receiver operating characteristic curve (ROC)—AUC with 95% CI, according to binomial exact distribution, was computed based on the four BI-RADS categories.

In order to establish a clinically and statistically acceptable threshold for accuracy of the breast density software system, the non-inferiority margin for the AUC was defined as the observed value for the routine evaluation made by radiologists in the Florence study. Since our outcome was a single ROC curve and the direct comparison with radiologist visual assessment was not possible, a non-inferiority margin was calculated based on the modification of confidence interval method suggested by Ahn et al.^22^. In the Florence study, consisting of 15,952 mammograms classified by radiologists’ visual assessment in the four BI-RADS categories, the AUC for predicting incident cases was 0.579 (Supplementary Fig. S1). The non-inferiority threshold was set at 10% reduction in area exceeding 0.5, if the lower bound of the 95% CI of AUC value for DSM was higher than 0.571.

Statistical analysis was performed using Stata version 10.0 (Stata Corporation, College Station, TX, USA).

### Power of the study

For the power calculation we used AUC of VolparaDensity’s automated system (version 3.1, Matakina Technology, Ely-Cambridgeshire, UK) from the Florence study as an expected value of software AUC. VolparaDensity is a commercial volumetric breast density method that operates on ‘for processing’ mammograms^23^. Expected value of the software AUC in the Florence study was 0.637 (re-analysis of data from Florence study)^17^. With an expected sample size of about 300 cancers and an underlying cohort of about 25,000 women aged 48–55, applying the same BI-RADS specific risk of cancer observed in the Florence study^17^, the power to exclude a non-inferiority threshold of 0.571 was expected to be over 99%.

## Results

In total, 14,267 mammograms from the same number of women, aged 48–55 years old, were available for density assessment, out of whom 322 had cancer (115 in RETomo, 63 in Florence study and 144 in PROCAS) (Table 1). Mean (SD) age of women in the pooled analysis was 51.0 ± 1.9 years old, while in RETomo it was 51.2 ± 2.1, in the Florence study 50.8 ± 1.4 and in PROCAS 51.3 ± 2.0.

**Table 1:** Distribution of cancer types and breast density categories among women by study centre.

	RETomo N = 8332	Florence N = 5359	PROCAS N = 576	Total N = 14,267
BI-RADS				
A	521 (6.3)	505 (9.4)	37 (6.4)	1063 (7.4)
B	3047 (36.6)	2237 (41.7)	208 (36.1)	5492 (38.5)
C	3701 (44.4)	2089 (39.0)	271 (47.1)	6061 (42.5)
D	1063 (12.7)	528 (9.9)	60 (10.4)	1651 (11.6)
Cancers	115	63	144	322
Prevalent cancers	41 (35.6)	39 (61.9)	144 (100.0)	224 (69.6)
Incident cancers	74 (64.4)	24 (38.1)	0 (0.0)	98 (30.4)
Interval cancers	26 (22.6)	9 (14.3)	0 (0.0)	35 (10.9)
Time from mammogram to cancer diagnosis* months, mean ± sd	24.5 ± 11.9	23.1 ± 5.4	NA	24.2 ± 10.7

*Only for incident cancers.

Out of 322 diagnosed cancers, 98 were incident round cases (74 in RETomo and 24 in Florence study), out of which 35 were interval cancers (26 in RETomo and 9 in Florence study) (Table 1). The distribution of BD was similar in all studies, with 7.4% in BI-RADS A and 11.6% in BI-RADS D (Table 1). In RETomo, women below 50 years old were referred to a 1-year interval, while those over 50 were referred to 2 years; BIRADS D was more frequent in women below 50 years old 15.9% vs. 11.1% compared to those over 50 years (Supplementary Table S2).

### Non-inferiority analysis

**Figure 2:**
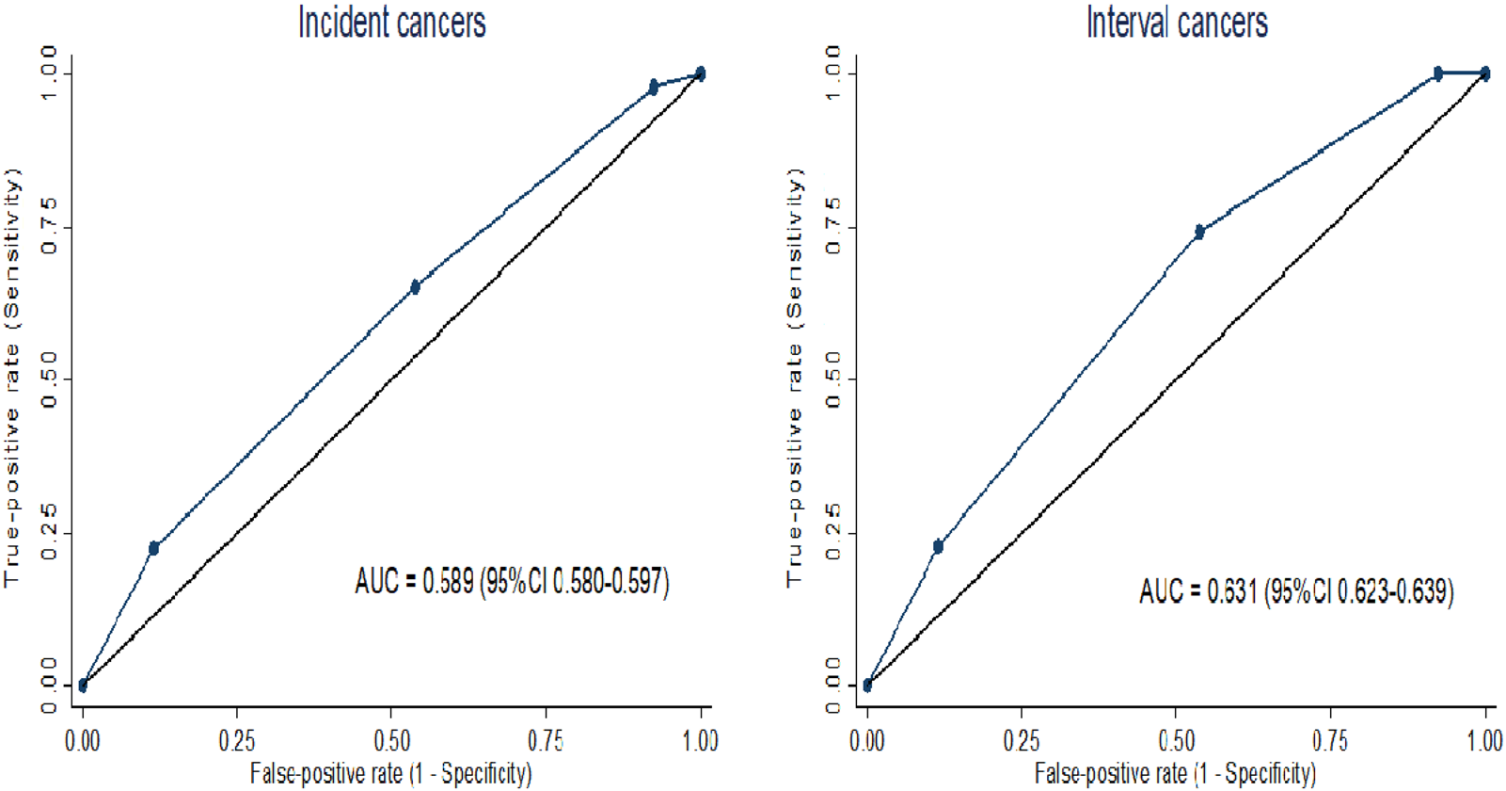
AUC for DenSeeMammo™ for incident and interval cancers (only RETomo and Florence).

Ability to predict risk for both incident (AUC = 0.589; 95% CI 0.580–0.597) and interval cancers (AUC = 0.631; 95% CI 0.623–0.639) was better for DSM than for radiologist visual assessment as both point estimates and lower bounds of 95% CI were above the predefined visual assessment threshold (0.571) (Fig. 2).

### Risk of breast cancer

The risk of incident cancer was almost three times higher for women in the highest category of breast density (BI-RADS D) than for those in the two lowest BI-RADS categories (OR 2.6, 95% CI 1.5–4.4), while the risk for interval cancer was almost four times higher for women in the abovementioned categories (OR 3.6; 95% CI 1.4–9.3) (Table 2). The risk for all cancers (OR 2.2; 95% CI 1.6–3.1) was similar to the risk for incident cancers, while risk for prevalent cancer was slightly lower (OR 1.8; 95% CI 1.2–2.6) (Table 3).

**Table 2:** Risk of incident and interval cancers according to the breast density as evaluated by DSM in RETomo and Florence studies.

BI-RADS	Women	Incident cancers		Interval cancers	
		Cancers	OR (95% CI)	Cancers	OR (95% CI)
A	1026 (7.5)	2 (2.0)		0 (0.0)	
B	5284 (38.6)	32 (32.7)	1.0	9 (25.7)	1.0
C	5790 (42.3)	42 (42.9)	1.4 (0.9–2.1)	18 (51.4)	2.2 (1.0–4.9)
D	1591 (11.6)	22 (22.4)	2.6 (1.5–4.4)	8 (22.9)	3.6 (1.4–9.3)
Total	13,691	98		35	

Values are n (%) unless otherwise stated.

**Table 3:** Risk of prevalent and all cancers according to the breast density as evaluated by DSM in all three study centres.

BI-RADS	Women	Prevalent cancers		All cancers	
		Cancers	OR (95% CI)	Cancers	OR (95% CI)
A	1063 (7.4)	10 (4.5)		12 (3.7)	
B	5492 (38.5)	74 (33.0)	1.0	106 (32.9)	1.0
C	6061 (42.5)	103 (46.0)	1.3 (1.0–1.8)	145 (45.0)	1.3 (1.0–1.6)
D	1651 (11.6)	37 (16.5)	1.8 (1.2–2.6)	59 (18.3)	2.2 (1.6–3.1)
Total	14,267	224		322	

When comparing the risk of incident cancer between the highest and lowest BI-RADS categories within each centre, the risk of cancer was similar in the Florence study (OR 2.6; 95% CI 0.8–8.7) and the RETomo study (OR 2.4; 95% CI 1.3–4.3) (Supplementary Table S3), with no evidence of heterogeneity.

## Discussion

In the present study we have evaluated the ability of a recently developed automated system for breast density assessment (DenSeeMammo) to predict risk of breast cancer in the pooled analysis of three European studies. Positive association of automatically assessed breast density and risk of incident, interval (masking effect) and prevalent breast cancers and all cancers was observed in the pooled data, and no important heterogeneity was observed among the studies.

Accuracy of DSM in breast cancer risk prediction was acceptable in our study with an AUC (0.589), not inferior to, and actually slightly higher than, the radiologist’s visual assessment observed in Florence^17^. It was also similar to those reported previously and estimated by using Volpara software^14,16,19^. Although these studies found positive associations of both automatically and visually assessed BD with breast cancer risk, their automated systems were inferior to BI-RADS visual assessment in the discrimination of cancer risk. Astley et al. also showed that the average of two radiologists estimates of breast density recorded on Visual Analogue Scales (VAS) was a stronger predictor for breast cancer, compared to four different automated systems^18^. In our study overall DSM accuracy outperformed the single radiologists’ visual breast density assessment, suggesting that DSM or other software with similar performance could be used in screening, thereby overcoming some of the organizational barriers that impede the radiologist’s visual assessment without the risk of decreasing accuracy.

This study did not aim to provide direct comparison with other automated software and Volpara was used as the only automated software available for the power calculation. Although a recent study demonstrated that DSM has a comparable or even better degree of agreement with expert radiologists’ visual assessment than Volpara^24^, the most studied and one of the best performing quantitative volumetric assessment method^14,18^, other studies performed on the same set of mammographic images with direct comparison with other automated systems are necessary to answer whether DSM is a valid alternative to other automated systems.

As expected, excess risk was higher for incident cancer (OR 2.6) than for overall cancers (OR 1.8) in our study. Data for incident cancers were less consistent between studies ranging from an OR of 2.8 in the PROCAS study^18^ and a HR of 8.3 in the study by Wanders et al.^25^. Considering only incident cases overestimates the true prediction performance of the software; of note is that both studies are case–control studies and for the latter, density was evaluated at the time of cancer detection, augmenting additionally the ability of the software to predict breast cancer. When evaluating risk of overall cancers, i.e., considering also prevalent cases, our results (OR 1.8) are in agreement with results of studies utilising other automatically assessed breast density software. In particular, prediction of cancer was very similar to the estimates of Puliti et al. who reported 2.0 times higher risk of invasive BC in women within the highest breast density category compared to those in the three lower categories^17^, as estimated by VolparaDensity software in the entire Florence cohort. It must be noted that only a small part of available cancers and mammograms were included in both of the density evaluation studies, because only one third of the Florence cohort used for validating Volpara software was also suitable for processing by DSM. With the limitation of the difference in age of the participants, screening frequency and breast density distribution, other studies reported similar results for the association between fully automatically assessed BD and risk of cancer, with OR ranging from 1.7 to 2.3 when using Volpara^14,15,18,19^, 1.9 and 3.9 Quantra^14,16^, 2.2 Densitas^18^ and 2.5 for a method using ImageJ^14^.

Association of breast density and risk of masking effect has been widely investigated due to the importance in patient risk stratification and identification of patients who will benefit from supplemental screening tests. However, only a few studies measured the association between automatically assessed BD and risk of interval cancer and estimates differs greatly, partly due to different choices of denominator (screen detected cancers, healthy women, or screen examinations). Odds ratio for interval cancer in our study (OR 3.6) was somewhat higher than that of Moshina et al. (OR 2.9)^26^ and lower than estimates of Destounis et al. (RR = 4.0)^27^, Wanders et al. (RR = 4.4 in the highest BD category vs. 0.7 in the lowest BD category)^28^ and Puliti et al. (RR = 5.0)^17^. It is hard to distinguish to what extent the interval cancer rate occurred truly due to masking and what part is attributed to rapidly evolving cancers, especially without information on size, grade and molecular subtypes of cancers. Nevertheless, our data and the previous literature clearly show that the proportion of interval cancers is higher in dense breasts than in fatty breasts^17,26–28^. One bias affecting our study is that part of the REtomo cohort, i.e., women aged 48–50, were invited for rescreening after 12 months. The chance of having an interval cancer in the first year after a negative screen is low and it is worth noting that dense breasts were slightly more prevalent in women that have been re-invited after 12 months (Supplementary Table S2). This bias could only underestimate the true contribution of the interval cancers to the overall masking effect in our study. Similarly, Holland et al. confirmed that automatically assessed percentage of dense breasts is associated with increased risk of interval breast cancer not only due to the breast density as a risk factor but also due to its ability to discriminate screen detected versus interval cancer, i.e., to capture masking effect^29^.

Our study has certain limitations which should be addressed. We defined the non-inferiority threshold using the radiologist’s visual assessment of breast density as standard. This was decided because routine evaluation by one radiologist is available in clinical practice in most screening programs. On the other hand, this standard is less accurate than that used in many studies, i.e., the judgement by a panel of radiologists; this latter option is a better gold standard with which to compare and has higher accuracy than what is usually available in routine practice. Furthermore, the threshold has been defined based on a single centre cohort, which may not be representative of performance in other centres. While in the PROCAS study women with breast cancer and controls were matched for major confounding variables, such as parity, body mass index and menopausal status, in the RETomo and Florence studies this was not possible, which hinders us from drawing conclusion about an association between BD and cancer risk and from generalising our results to other centres. Although the software works on “for presentation” images and is multivendor, not all the stored images were suitable for evaluation.

Both the metrics which we used for evaluation (AUC and OR computed in logistic regression) are very sensitive to the breast density threshold used. The DSM adopted threshold is calibrated to reproduce the distribution of BI-RADS categories in the general screening population^20^, but not to optimize risk prediction. This choice guarantees comparability with previous studies and makes meaningful the choice of the non-inferiority threshold but could be reconsidered when applying BD for risk stratification, because continuous values contain valuable additional information.

Finally, our study included only women of peri-menopausal age, when BD changes rapidly, decreasing in most women as they age; how much the predictivity observed in this age group can be generalised to older women should be assessed in a further study. Another potential selection bias could have been introduced by selecting only available images (for Florence, only the images from one vendor were available for processing, and in RETomo, only few images collected at the very beginning of the study were not stored correctly in order to be processed). Although this selection did not occur at random, these reasons are less likely to be associated with breast density and outcome in a population-based screening programme.

## Conclusions

DenSeeMammo, a new fully automated software for breast density assessment, is non inferior to radiologist’s visual assessment in the prediction of breast cancer risk. Automatically assessed breast density was strongly associated with incident and more strongly with interval cancers, indicating the ability to capture the masking effect of BD.

## Supplementary Information

Supplementary Information.

## References

[1] Sprague BL, Conant EF, Onega T, Garcia MP, Beaber EF, Herschorn SD (2016). Variation in mammographic breast density assessments among radiologists in clinical practice: A multicenter observational study. Ann. Intern. Med..

[2] American College of Radiology. *ACR Statement on Reporting Breast Density in Mammography Reports and Patient Summaries* (2017). https://www.acr.org/Advocacy-and-Economics/ACR-Position-Statements/Reporting-Breast-Density. Accessed 7 Sept 2021.

[3] Holland K, van Zelst J, den Heeten GJ, Imhof-Tas M, Mann RM, van Gils CH (2016). Consistency of breast density categories in serial screening mammograms: A comparison between automated and human assessment. Breast.

[4] Sickles E, D’Orsi CJ, Bassett LW, D’Orsi CJ, Mendelson EB, Ikeda DM (2003). ACR BI-RADS mammography, Atlas. ACR Breast Imaging Reporting and Data System.

[5] Vourtsis A, Berg WA (2019). Breast density implications and supplemental screening. Eur. Radiol..

[6] Bakker MF, de Lange SV, Pijnappel RM, Mann RM, Peeters PHM, Monninkhof EM (2019). Supplemental MRI screening for women with extremely dense breast tissue. N. Engl. J. Med..

[7] Paci E, Mantellini P, Giorgi Rossi P, Falini P, Puliti D, TBST Working Group (2013). Tailored breast screening trial (TBST). Epidemiol. Prev..

[8] Esserman LJ, WISDOM Study and Athena Investigators (2017). The WISDOM study: Breaking the deadlock in the breast cancer screening debate. NPJ Breast Cancer.

[9] National Library of Medicine (US). *Identifier NCT03672331. My Personalized Breast Screening (MyPeBS)* (2021). https://clinicaltrials.gov/ct2/show/NCT03672331?cond=My+Personalized+Breast+Screening&draw=2&rank=1. Accessed 9 Sept 2021.

[10] Sartor H, Lång K, Rosso A, Borgquist S, Zackrisson S, Timberg P (2016). Measuring mammographic density: Comparing a fully automated volumetric assessment versus European radiologists’ qualitative classification. Eur. Radiol..

[11] Ciatto S, Bernardi D, Calabrese M, Durando M, Gentilini MA, Mariscotti G (2012). A first evaluation of breast radiological density assessment by QUANTRA software as compared to visual classification. Breast.

[12] Richard-Davis G, Whittemore B, Disher A, Rice VM, Lenin RB, Dollins C (2018). Evaluation of quantra hologic volumetric computerized breast density software in comparison with manual interpretation in a diverse population. Breast Cancer (Auckl.).

[13] Keller BM, Chen J, Daye D, Conant EF, Kontos D (2015). Preliminary evaluation of the publicly available laboratory for breast radiodensity assessment (LIBRA) software tool: Comparison of fully automated area and volumetric density measures in a case-control study with digital mammography. Breast Cancer Res..

[14] Eng A, Gallant Z, Shepherd J, McCormack V, Li J, Dowsett M (2014). Digital mammographic density and breast cancer risk: A case-control study of six alternative density assessment methods. Breast Cancer Res..

[15] Brand JS, Czene K, Shepherd JA, Leifland K, Heddson B, Sundbom A (2014). Automated measurement of volumetric mammographic density: A tool for widespread breast cancer risk assessment. Cancer Epidemiol. Biomark. Prev..

[16] Brandt KR, Scott CG, Ma L, Mahmoudzadeh AP, Jensen MR, Whaley DH (2016). Comparison of clinical and automated breast density measurements: Implications for risk prediction and supplemental screening. Radiology.

[17] Puliti D, Zappa M, Giorgi Rossi P, Pierpaoli E, Manneschi G, Ambrogetti D (2018). Volumetric breast density and risk of advanced cancers after a negative screening episode: A cohort study. Breast Cancer Res..

[18] Astley SM, Harkness EF, Sergeant JC, Warwick J, Stavrinos P, Warren R (2018). A comparison of five methods of measuring mammographic density: A case-control study. Breast Cancer Res..

[19] Jeffers AM, Sieh W, Lipson JA, Rothstein JH, McGuire V, Whittemore AS (2017). Breast cancer risk and mammographic density assessed with semiautomated and fully automated methods and BI-RADS. Radiology.

[20] Balleyguier C, Arfi-Rouche J, Boyer B, Gauthier E, Helin V, Loshkajian A (2019). A new automated method to evaluate 2D mammographic breast density according to BI-RADS® Atlas fifth edition recommendations. Eur. Radiol..

[21] Pattacini P, Nitrosi A, Giorgi Rossi P, Iotti V, Ginocchi V, Ravaioli S (2018). Digital mammography versus digital mammography plus tomosynthesis for breast cancer screening: The Reggio Emilia tomosynthesis randomized trial. Radiology.

[22] Ahn S, Park SH, Lee KH (2013). How to demonstrate similarity by using noninferiority and equivalence statistical testing in radiology research. Radiology.

[23] Highnam R, Brady M, Yaffe MJ, Karssemeijer N, Harvey J, Martí J, Oliver A, Freixenet J, Martí R (2010). Robust breast composition measurement—VOLPARA^TM^. Digital Mammography.

[24] Weigert JMJ, Helin V, Villejuif FR (2019). Use of a new 2D tool to estimate breast density and evaluate its use in a breast cancer risk calculation model for risk stratification. ECR 200019 Book Abstr..

[25] Wanders JOP, Holland K, Karssemeijer N, Peeters PHM, Veldhuis WB, Mann RM (2017). The effect of volumetric breast density on the risk of screen-detected and interval breast cancers: A cohort study. Breast Cancer Res..

[26] Moshina N, Sebuødegård S, Lee CI, Akslen LA, Tsuruda KM, Elmore JG (2018). Automated volumetric analysis of mammographic density in a screening setting: Worse outcomes for women with dense breasts. Radiology.

[27] Destounis S, Johnston L, Highnam R, Arieno A, Morgan R, Chan A (2017). Using volumetric breast density to quantify the potential masking risk of mammographic density. Am. J. Roentgenol..

[28] Wanders JO, Holland K, Veldhuis WB, Mann RM, Pijnappel RM, Peeters PH (2017). Volumetric breast density affects performance of digital screening mammography. Breast Cancer Res. Treat..

[29] Holland K, van Gils CH, Mann RM, Karssemeijer N (2017). Quantification of masking risk in screening mammography with volumetric breast density maps. Breast Cancer Res. Treat..

