# Supplementary material for: Validation of a new fully automated software for 2D digital mammographic breast density evaluation in predicting breast cancer risk
Source: Sci Rep. 2021 Oct 6;11:19884. doi: 10.1038/s41598-021-99433-3 (PMC8494838; doi:10.1038/s41598-021-99433-3)

**SUPPLEMENTARY MATERIAL**

**Supplementary Table 1** Summary of studies included in the analysis

|  | **RETomo^24^** | **Florence study^20^** | **PROCAS^21^** |
| --- | --- | --- | --- |
| **Study design** | Cohort | Cohort | Nested Case-Control in a Cohort |
| **Study setting** | Province of Reggio Emilia | Province of Tuscany | Greater Manchester |
| **Study period** | March 2014- July 2017 | 2006–2013 | October 2009 -March 2015 |
| **No of women in the study** | 27.000 | 15.952 | 57.905 |
| **Screening policies** | **Since**: 1999  **Target population**: women aged 45–74 years who are actively invited by the screening programme by appointment call and recall system  **Screening interval**: 1 year for women aged 45-49 and 2 years for women aged 50-74 | **Since**: 1991  **Target population:** women aged 50–69 years  **Screening interval**: 2 years | **Since:** 1988  **Target population:** women aged 50–69 years. Women who have family histories of breast cancer can obtain enhanced screening from the age of 40 years.  The Age Extension study included women aged 47-49 and 70-73 years  **Screening interval**: 3 years |
| **Participants** | Women aged 45–70 attending screening in one of three clinics equipped with DBT and who had already participated in at least one round of the Reggio Emilia screening programme | All women who performed the first screening digital mammography in the age class 49-54 years during the period 2006-2013 | All women invited and consented to take part in the Greater Manchester Breast Screening Service for routine 3-year mammographic screening between October 2009 and March 2015 |
| **Screening round** | All have previous screening round | First screening round | All women attending screening from Oct 2009–Oct 2012, after Oct 2012 only women attending their first (prevalent) screen were invited.  For the purposes of this study it was women who were detected with cancer at their screen on entry to PROCAS. |
| **Inclusion criteria** | Women aged 45–70 attending screening in one of three clinics equipped with DBT and who had already participated in at least one round of the Reggio Emilia screening programme | All women who performed the first screening digital mammography in the age class 49-54 years during the period 2006-2013 | All women in the case-control study had to have GE digital screening mammograms with raw (‘for processing’) image data as well as BD assessment by two radiologists recorded on VAS |
| **Exclusion criteria** | Women with previous breast cancer, prior DBT, and ascertained genetic risk for breast cancer (based on a standardized risk score used by the regional screening program), women with very large breasts,  a familial risk score updated due to recent breast cancer in relatives, augmentation prostheses, pregnancy or technical reasons, i.e., randomization procedure or the tomosynthesis was temporarily unavailable | -they had had previous BC;  - they had breast implants at the time of first DM (breast implants impair BD measurement and mammography sensitivity);  - they had previously enrolled in the active arm of an Italian study^23^ offering ultrasound in addition to screening mammography | All women with a previous diagnosis of breast cancer |
| **Endpoints** | Interval cancers and cumulative incidence of advanced cancers (≥ 20 mm diameter) in the 4.5 years after recruitment. | – Interval cancers at the first round, i.e., clinically detected after the first negative screening episode  (negative mammogram or positive mammogram that did not lead to cancer detection) and before the date of the second invitation  – Screen-detected at the second round  – Advanced cancer after a negative screening episode (i.e., cancers diagnosed at stage II or more advanced during the screening interval or at the subsequent round) and the total of women screened negative | Occurrence of a breast cancer by June 2014 |
| **Breast cancer ascertainment** | Biopsy | Linkage with Tuscan cancer registry | 1. Matching the PROCAS data set to the Somerset Cancer Registry.  2. Matching with the NWCIS database, a histological database of breast cancer diagnoses, for cancers diagnosed in the northwest.  3. Notifications from participants that they had been diagnosed with breast cancer. |
| **Mammography/Tomosynthesis** | Mammogram readings performed by ten radiologists whose screening experience ranges from 4 to 20 years. All fulfil the regional screening quality assurance criterion of at least 5000 mammograms read per year and participate in periodic audits reviewing individual performance indicators and interval cancer imaging review. DBT was introduced 18 months before the start of the trial and was used for all symptomatic women and assessment |  | Density was assessed using all mammographic views of the contralateral breast in cases and matched breast in controls. Mammograms were mainly analogue in the initial 12-month period (October 2009–September 2010), switching to completely digital thereafter (October 2010–June 2014). The date of entry to the study was the date of mammogram at study entry |
| **Image acquisition** | 11 GE Senographe Essential digital systems (GE Healthcare, Buc, France) four of which are equipped with tomosynthesis | GE (Senographe essential) and Siemens (Mammomat Inspiration) images.  Only Siemens images can be processed by DenSeeMammo (5359 women, 63 cancers) | Two different types of FFDM system [Fischer Senoscan (Carestream Health Inc., Rochester, NY, USA) and GE Essential (GE Medical Systems Ltd, Chalfont St Giles, UK)].  In the case-control study, all with GE Essential |
| **Density evaluation** | DenSeeMammo^TM^ | DenSeeMammo^TM^  Volpara  Single radiologist routine | DenSeeMammo^TM^  Volpara  VAS of the radiologists’ panel |
| **Image management** | PACS system | PACS system | PACS system |

BD, breast density, DBT, digital breast tomosynthesis; DM, digital mammography; FFDM, full-field digital mammography; VAS, visual analog scale; NWCIS, The North West Cancer Intelligence Service; PACS, picture archiving communications system.

**Supplementary Table 2** Distribution of breast density categories in RETomo and Florence study

| **BI-RADS** | **ReTOMO** | | |  | **Florence study** |
| --- | --- | --- | --- | --- | --- |
|  | Total | <50 years  (1 year screening interval) | ≥50 years  (2 years screening interval) |  | 49-54 years  (2 years screening  interval) |
| **A** | 521 | 134 (4.7) | 387 (7.1) |  | 505 (9.4) |
| **B** | 3047 | 853 (29.7) | 2194 (40.2) |  | 2237 (41.7) |
| **C** | 3701 | 1428 (49.7) | 2273 (41.6) |  | 2089 (39.0) |
| **D** | 1063 | 459 (15.9) | 604 (11.1) |  | 528 (9.9) |
| **Total** | 8332 | 2874 | 5458 |  | 5359 |

**Supplementary Table 3** Risk of incident breast cancer according to the breast density categories as evaluated by automated system DenSeeMammo in ReTomo and Florence studies

| **BI-RADS** | **RETomo** | | |  | **Florence** | | |
| --- | --- | --- | --- | --- | --- | --- | --- |
|  | Women | Cancers | OR (95% CI) |  | Women | Cancers | OR (95% CI) |
| **A** | 521 (6.3) | 1 (1.4) | 1.0 |  | 505 (9.4) | 1 (4.2) | 1.0 |
| **B** | 3047 (36.6) | 25 (33.8) |  |  | 2237 (41.7) | 7 (29.2) |  |
| **C** | 3701 (44.4) | 30 (40.5) | 1.1 (0.6-1.9) |  | 2089 (39.0) | 12 (50.0) | 2.0 (0.8-4.8) |
| **D** | 1063 (12.7) | 18 (24.3) | 2.4 (1.3-4.3) |  | 528 (9.9) | 4 (16.7) | 2.6 (0.8-8.7) |
| **Total** | 8332 | 74 |  |  | 5359 | 24 |  |

Values are N (%) unless otherwise stated.

**Supplementary Table 4** Risk of prevalent breast cancer according to the breast density categories as evaluated by automated system DenSeeMammo in all three studies

| **BI-RADS** | **RETomo** | | |  | **Florence** | | |  | **PROCAS** | | |
| --- | --- | --- | --- | --- | --- | --- | --- | --- | --- | --- | --- |
|  | Women | Cancers | OR (95% CI) |  | Women | Cancers | OR (95% CI) |  | Women | Cancers | OR (95% CI) |
| **A** | 521 (6.3) | 1 (2.5) | 1.0 |  | 505 (9.4) | 4 (10.3) | 1.0 |  | 37 (6.4) | 5 (3.5) |  |
| **B** | 3047 (36.6) | 11 (26.8) |  |  | 2237 (41.7) | 16 (41.0) |  |  | 208 (36.1) | 47 (32.6) | ref. |
| **C** | 3701 (44.4) | 19 (46.3) | 1.53 (0.74-3.15) |  | 2089 (39.0) | 17 (43.6) | 1.12 (0.58-2.14) |  | 271 (47.1) | 67 (46.5) | 1.22 (0.81-1.84) |
| **D** | 1063 (12.7) | 10 (24.4) | 2.81 (1.21-6.53) |  | 528 (9.9) | 2 (5.1) | 0.52 (0.12-2.22) |  | 60 (10.4) | 25 (17.4) | 2.65 (1.46-4.82) |
| **Total** | 8332 | 41 |  |  | 5359 | 39 |  |  | 576 | 144 |  |

**Supplementary Figure 1.** Unpublished data from Florence study used for non-inferiority threshold calculation and power calculation


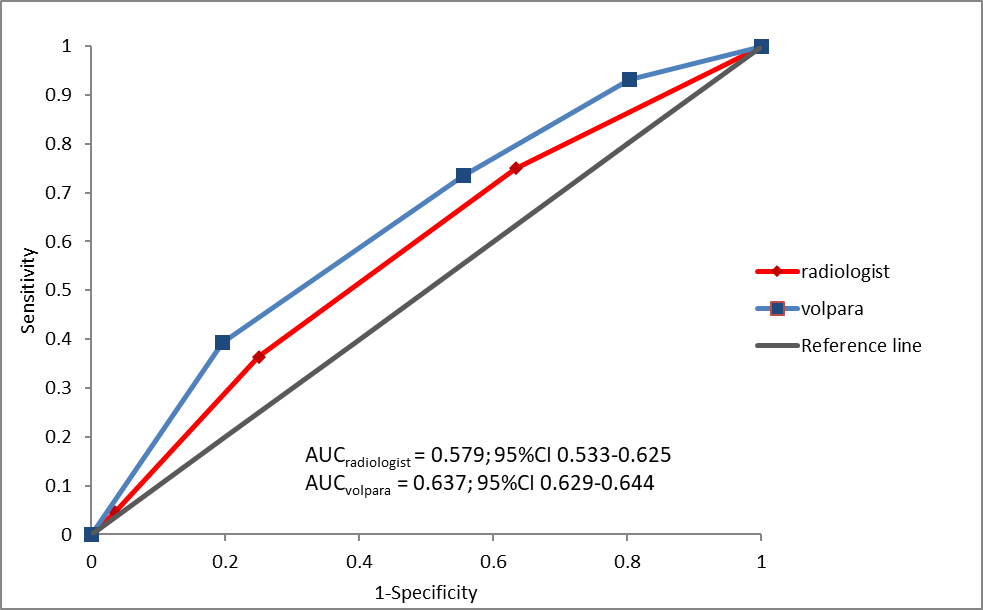


**Supplementary Figure 2.** AUC for DenSeeMammo™ in prediction of incident (A) and interval (B) cancers by study centre


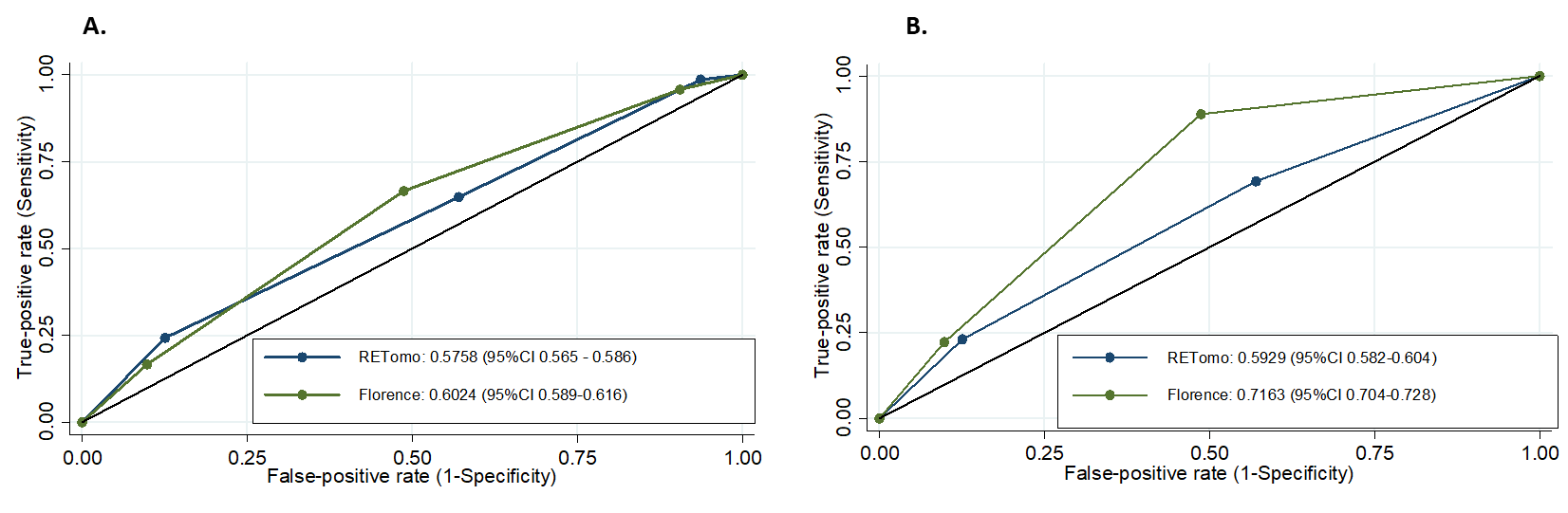


**Supplementary Figure 3**. AUC for DenSeeMammo™ in prediction of all cancers by study centre


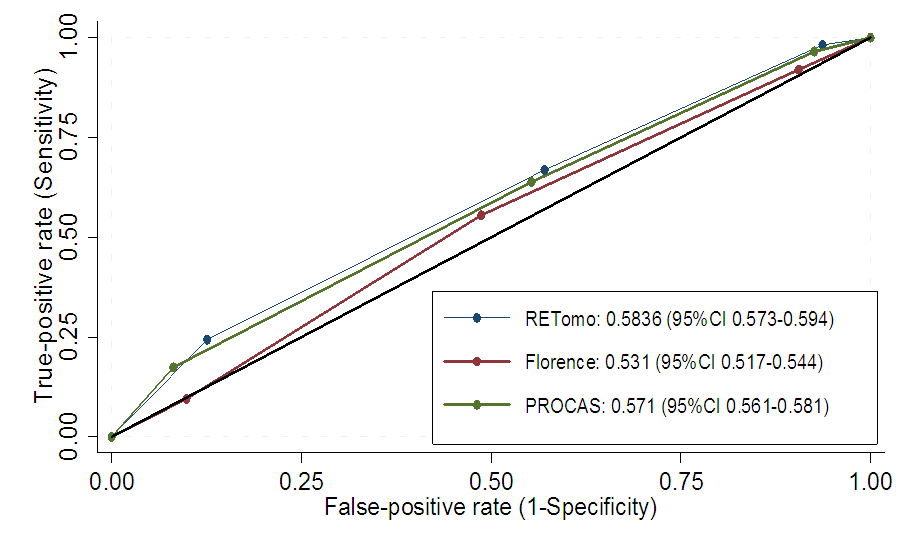

Supplement: Supplementary file 1 — Supplementary Information. [file 41598_2021_99433_MOESM1_ESM.docx]
